# Harnessing the Power of Mucosal-Associated Invariant T (MAIT) Cells in Cancer Cell Therapy

**DOI:** 10.3390/biomedicines10123160

**Published:** 2022-12-07

**Authors:** Chie Sugimoto, Hiroyoshi Fujita, Hiroshi Wakao

**Affiliations:** Host Defense Division, Center for Frontier Medicines, Dokkyo Medical University, Mibu 321-0293, Japan

**Keywords:** MAIT cells, iPSC, reMAIT cells, immune cell therapy, adoptive transfer, off-the-shelf, tumor

## Abstract

Mucosal-associated invariant T (MAIT) cells, a burgeoning type of the innate-like T cells, play a crucial role in maintaining immune homeostasis, particularly in host defense. Although many studies have implied the use of MAIT cells in tumor immunity, whether MAIT cells are pro-tumor or anti-tumor has remained elusive, as in the case for other innate-like T cells that possess dichotomous roles in tumor immunity. Although this difficulty persists where endogenous MAIT cells are the target for therapeutic intervention, the advent of induced pluripotent stem-cell-derived MAIT cells (reMAIT cells) will make it possible to harness these cells for immune cell therapy. In this review, we will discuss possible roles of MAIT cells in tumor immunity and the potential of reMAIT cells to treat tumors.

## 1. Introduction

Mucosal-associated invariant T (MAIT) cells are a type of innate-like T cell (also known as unconventional T cells) and are abundantly present in human blood, intestinal mucosa, liver, and lung tissue. They are also found in tumor infiltrating lymphocytes (TIL) across a wide array of tumors [1,2]. In addition to conventional T cells (CD4 and CD8 T cells), innate-like T cells, including MAIT cells, natural killer T (NKT) cells, and γδT cells, have been shown to play a pivotal role in tumor immunity, raising the possibility of using or targeting these cells in tumor therapy [3]. Nevertheless, due to their dichotomic function (pro-tumor and anti-tumor), harnessing innate-like T cells for tumor therapy requires further knowledge of these cells. Indeed, many contradicting results have been reported with innate-like T cells, which has made it difficult to use them as they stand [4,5]. It is thus desirable to control innate-like T cells to exert only anti-tumor activity when harnessing the cells for tumor therapy. Our recent report explored such a concept and suggested the possibility of induced pluripotent stem-cell (iPSC)-derived MAIT cells in tumor immune cell therapy or prophylaxis to prevent relapse [6]. In this review, we will discuss current knowledge regarding the role of MAIT cells in tumor immunity and the possible use of iPSC-derived MAIT cells combined with chimeric antigen receptor (CAR) technology for exploring future immune cell therapy.

## 2. General Features of MAIT Cells

MAIT cells play a pivotal role in immunity. Indeed, MAIT cells are implicated in cancers as well as in an array of diseases such as bacterial, viral, and parasitic infections, and allergic, autoimmune, and metabolic diseases [7]. As is the case for other innate-like T cells, MAIT cells are characterized for the semi-invariant T cell receptor (TCR) alpha variable (TRAV)—1-2-TCR alpha joint (TRAJ)-33, -12, and -20 for human and TRAV1-TRAJ33 for mouse—and for the biased use of TCR beta repertoires [8,9]. An early study identified CD161 (killer cell lectin-like receptor), CD26 (dipeptidyl peptidase IV), and CD243 (ATP-dependent multidrug efflux pump) as the markers for MAIT cells [1]. MAIT cells can express activation markers (CD44, CD25, CD38, CD39, CD69 and human leukocyte antigen (HLA)-DR) upon activation. Furthermore, MAIT cells harbor the costimulatory/coinhibitory molecules, CD27, CD28, programmed cell death receptor (PD)-1, and cytotoxic T-lymphocyte-associated antigen (CTLA-4) [10,11,12,13], the chemokine receptors, CCR5, CCR6, CCR9, and CXCR6, and the cytokine receptors, interleukin (IL)-1 receptor (R), IL-7R, IL-12R, IL-15R, IL-18R, and IL-23R [7]. MAIT cells are, however, unique in terms of transcription factor expression. In fact, MAIT cells could harbor promyelocytic leukemia zinc finger (PLZF), retinoic acid receptor-related orphan receptor (ROR)γt, T-bet, and Eomesdermin (Eomes). PLZF is required for functional maturation and for exerting the innate-like features of MAIT cells, such as immediate production of inflammatory cytokines and cytotoxic molecules. Whereas RORγt is required for the development of T helper (Th) 17 cells and of group 3 innate lymphoid cells (ILC3s) [14], T-bet and Eomes are required for Th1 development and the cytotoxic activity of NK cells and CD8 T cells, respectively [15].

Two subsets of MAIT cells are known: MAIT1 cells and MAIT17 cells, which are characterized by the production of IFN-γ and L-17, respectively. Whereas MAIT17 cells are characterized by expression of RORγt, T-bet expression in ΜAΙΤ1 cells distinguishes their ability to produce IFN-γ. In mice, MAIT17 cell are dominant over MAIT1 cells [16]. 

Development of MAIT cells is contingent upon non-polymorphic major histocompatibility complex I-related molecule (MR1) and microbiota, and it consists of three stages as defined by the expression of CD24 and CD44 (stage 1–3), in which stage 3 consists of extrathymic expansion and maturation [17,18]. MR1 presents the non-peptide antigens: vitamin B2 metabolites, such as 5-(2-oxoethylideneamino)-6-D-ribitylaminouracil (5-OE-RU) and 5-(2-oxopropylideneamino)-6-D-ribitylaminouracil (5-OP-RU), and vitamin B6 metabolites, such as 6-formyl pterin (6-FP). Whereas 5-OE-RU and 5-OP-RU function as agonists, 6-FP antagonizes the function of MAIT cells [19,20]. Development of MAIT cells in the murine thymus is dependent on 5-OP-RU synthesized by bacteria harboring the vitamin B2 biosynthesis pathway [21]. Accordingly, germ-free mice lack MAIT cells [17,22].

There exist two different pathways for MAIT cell activation, one dependent on TCR and the other one independent of TCR. The former is triggered through the presentation of antigens, such as 5-OE-RU and 5-OP-RU, loaded onto MR1 in most, if not all, cells and is not limited to professional antigen presenting cells (APCs) such as dendritic cells, macrophages, and B cells. Such TCR engagement results in activation of MAIT cells, as manifested by upregulation of CD25, CD69, CD38, and HLA-DR, followed by TNF-α and/or IL-17 production [1,19]. MAIT cells are also activated by the cytokines IL-12, IL-15, and IL-18, which are TCR-independent in nature [23]. Intriguingly, TCR-dependent activation results in more production of inflammatory cytokines than TCR-independent activation [24].

## 3. MAIT Cells in Human Cancer

The first report implying the involvement of MAIT cells in tumors was based on the presence of the transcripts pertinent to MAIT cells, such as *TRAV1-2-TRAJ33*, in kidney and brain tumors [25]. Since that report, the involvement of MAIT cells in tumors has been suggested by the quantitative and qualitative analyses of MAIT cells in peripheral blood and/or in tumors. Nonetheless, whether MAIT cells are pro-tumor or anti-tumor is still a target for intensive scrutiny and the answer has yet to be deciphered. Below, we briefly summarize the pro- and anti-tumor features of MAIT cells in human cancers. Readers interested in a more detailed description of MAIT cells in individual tumors, please refer to Cogwell et al. [11].

## 4. Pro-Tumor and Anti-Tumor Functions of MAIT Cells in Mucosal Cancers

Given the intimate interaction between MAIT cells and the microbiome, many studies have focused on the role(s) of MAIT cells in mucosal tumors, such as colorectal cancer (CRC), hepatocellular carcinoma (HCC), and lung cancers. In CRC, the MAIT cells (defined as CD3^+^CD26^+^Vα7.2^+^ cells) increase in tumor sites is concomitant with a poor prognosis, suggesting that MAIT cells are tumor-promoting [26]. Similar increases in MAIT cells at tumor sites, relative to adjacent healthy tissue, and an accompanying decrease in MAIT cells in the periphery has been reported in CRC [27]. Although accumulation of MAIT cells with Th1 phenotype is seen in CRC tumor sites concomitant with expression of granzyme B, a protein pertinent to cytotoxic activity, whether these MAIT cells exert pro- or anti-tumor function remains obscure [28]. Contrary to these reports, Ling et al. demonstrated that MAIT cells inhibited cell cycle progression in tumor cells in a cell–cell contact-dependent manner, implying that MAIT cells are anti-tumor [29]. In HCC patients, MAIT cells decrease in tumors relative to healthy tissue and such a decrease correlates with poor prognosis, implying that MAIT cells could be anti-tumor [30]. In contrast, increases in activated and exhausted MAIT cells in tumors correlates with poor prognosis, suggesting that MAIT cells are pro-tumor in HCC [31]. One reasonable interpretation of such contradicting data is that the former used only RNA-sequencing for analysis of MAIT cells, while the latter addressed the phenotypes as well as the transcriptomes of MAIT cells. Upregulation of immune checkpoint proteins, such as PD-1, CTLA-4, and T-cell immunoglobulin mucin-3 (TIM-3), and of transcripts *IL8* and *CXCL12,* concomitant with downregulation of granzyme B and perforin in tumor MAIT cells, indicates that MAIT cells are exhausted and pro-tumor in HCC [31].

Cholangiocarcinoma (CCA) is a rare malignancy of the bile duct, representing around 3% of gastro-intestinal tumors. In CCA, a high MAIT-cell number in the tumor positively correlates with prolonged survival. The increase in MAIT cells in CCA correlates with a favorable anti-tumor immune signature, reflecting various immune steps towards anti-tumor activity, such as cancer antigen presentation, priming and activation of immune cells, and recognition of tumor cells by T cells [32]. Nonetheless, whether MAIT cells per se exert direct anti-tumor activity through recognition of tumor antigen(s) and cytotoxic activity has yet to be determined. 

## 5. Pro-Tumor and Anti-Tumor Functions of MAIT Cells in Other Cancers

Multiple myeloma (MM) is a hematological tumor that features a clonal expansion of malignant plasma cells in the bone marrow. In MM, decrease in MAIT-cell frequency in the peripheral blood as well as MAIT-cell dysfunction have been reported. Contrary to other inflammatory diseases in which MAIT cells converge at the inflammatory site, concomitant with a decline in the periphery, no accumulation of MAIT cells is found in the bone marrow. Moreover, MAIT cells from healthy donors detect 5-OP-RU-loaded MM cell lines and kill them with similar kinetics to NK cells in vitro [33]. 

Single cell RNA-sequencing analysis of TIL across the different tumors uncovered the cluster IL-17 producing T (Tc17) in which almost the half consisted of MAIT cells [2]. Given that TILs are often compromised for anti-tumor effector functions due to upregulation of the immune checkpoint proteins PD-ligand (L)1, V domain-containing Ig suppressor of T-cell activation (VISTA), TIM-3 and CTLA-4, it is important to elucidate whether immune checkpoint inhibitors (ICHs) also reinvigorate the anti-tumor function(s) of MAIT cells in addition to conventional T cells and NK cells. Such knowledge would further boost the therapeutic efficacy of ICHs. 

## 6. MAIT Cells and Microbiota in Cancer

Given that MAIT cells recognize bacteria-borne antigens, it is possible that bacteria present in the tumor microenvironment affect the phenotype and/or functions of MAIT cells. Several reports have shown such a possible link. Whereas breast duct-derived MAIT cells produce IFN-γ and IL-17 upon PMA/ionomycin stimulation, stimulation of a breast carcinoma cell line loaded with *Escherichia coli* results in only IL-17 production, implying that bacteria could shape the immune function of MAIT cells [34]. The activation status of MAIT cells in tumors is also affected by bacteria. Among CRC, non-small-cell lung carcinoma and renal-cell carcinoma, only MAIT cells derived from TILs in CRC show enhanced CD39, an activation marker that appears to be *Fusobacterium nucleatum* and TCR-dependent [35].

## 7. MAIT Cells in Murine Cancer Models

**Figure 1:**
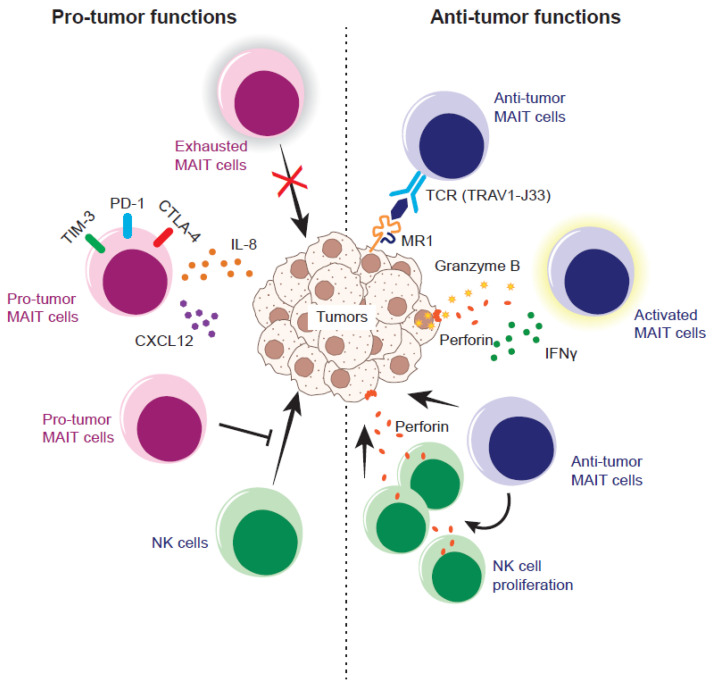
Pro- and anti-tumor nature of MAIT cells in the murine tumor model. Exhausted MAIT cells fail to control tumor growth and/or pro-tumor MAIT cells instigate tumor growth and/or metastasis by secreting IL-18 and/or CXCL12. Pro-tumor MAIT cells also inhibit the anti-tumor function of NK cells through suppressing the production of IFN-γ and degranulation. In contrast, anti-tumor MAIT cells recognize the ligand (5-OP-RU and/or tumor-derived ligands) loaded onto MR1 on the tumor cells via a T cell receptor (TCR) and stimulate the proliferation of NK cells armed with an effector molecule, perforin, which, in turn, results in tumor lysis. Alternatively, anti-tumor MAIT cells could recognize the tumor cell in a TCR-independent fashion and lyse the tumor cells.

Similar to studies in humans, conflicting results have been obtained regarding the functions of MAIT cells in murine cancer models. Here, we briefly summarize the pertinent data (Figure 1).

One study demonstrated that MAIT cells are pro-tumor in tumor initiation, growth, and experimental lung metastasis in vivo, even though MAIT cells possess tumor-killing potential in vitro [36]. MR1 knockout mice (therefore without MAIT cells) are resistant to metastasis of inoculated B16F10 melanoma, which is dependent on NK cells. Similarly, MR1 knockout mice exhibit tougher, resistance relative to the control mice, upon methylcholanthrene (a tumor-initiating reagent) inoculation. Furthermore, inoculation of 5-OP-RU pulsed-B16F10 exacerbates lung metastasis in a MAIT-cell-dependent manner. The pro-tumor mechanism in this case is that MAIT cells suppress NK cell effector functions, such as IFN-γ production and degranulation, which are prerequisites for NK cells to exert cytotoxic activity. Another report, using B6-MAIT_Cast_ mice which harbor an increased frequency of MAIT cells [37], shows the pro-tumor effects of MAIT cells, as evidenced by accelerated tumor growth and shortened survival in subcutaneous B16F10 inoculation relative to B6-MAIT_Cast_MR1^-/-^ mice devoid of MAIT cells [38]. Depletion of NK cells from B6-MAIT_Cast_MR1^-/-^ mice results in a compromised anti-tumor immune response, further demonstrating the pivotal role of NK cells in the anti-tumor response in the mice. 

However, the same study also indicated that activation of MAIT cells converts them from pro-tumor to anti-tumor. That is, pulsing B16F10 with 5-OP-RU prior to inoculation leads to suppression of metastasis in B6-MAIT_Cast_ mice but not in B6-MAIT_Cast_MR1^-/-^ mice. In a similar context, intranasal instillation of 5-OP-RU prior to tumor inoculation leads to increase in MAIT cells, which in turn results in a further decrease in B16F10 and human breast cancer cell-line-E0771 metastases in B6-MAIT_Cast_ mice but not in B6-MAIT_Cast_MR1^-/-^ mice, again arguing that TCR-activated MAIT cells are crucial for the anti-tumor immune response. The 5-OP-RU-dependent expansion and activation of MAIT cells induces a stronger expansion of NK cells with anti-tumor signatures, such as IFN-γ production and enhanced NKG2D and KLRG1 expression. However, how long the tumor-inhibitory response of such MAIT cells persists, and ultimately leads to prolonged survival of mice remains unclear because there is no guarantee that decreased metastasis at the defined time point always engenders the prolongation of survival. Nevertheless, the authors also showed that the proliferation of NK cells from melanoma patients and the production of IFN-γ ex vivo are boosted by MAIT cells in the presence of 5-OP-RU, further underpinning the importance of the MAIT–NK cell axis in human tumor immunity [38]. 

The anti-tumor nature of MAIT cells is further supported by the work of Ruf et al. [39]. MAIT cells proliferated in vivo upon administration of 5-OP-RU and CpG, a ligand for Toll-like receptor (TLR) 9, inhibited metastasis of B16F10 and RIL-175, a murine hepatocellular carcinoma cell line, and improved mouse survival upon intrahepatic B16F10 inoculation. However, the same did not happen in MR1 knockout mice [39]. Furthermore, tumor inoculation followed by 5-OP-RU and CpG administration results in enhanced infiltration of MAIT cells into the tumor concomitant with an increase in NK cells armed with perforin, an effector molecule required for cytotoxicity by pore formation on the target cell plasma membrane. This also implies the importance of NK cells in anti-tumor immunity, although there are no experiments to demonstrate their direct involvement by depleting NK cells in mice. Importantly, such MAIT-cell-mediated anti-tumor activity is independent of MR1 expression on tumor cells, implying that tumor recognition by MAIT cells via cognate MR1–TCR interaction may not be required. Such an MR1-independent anti-tumor immune response is of importance, given that not all tumors necessarily express MR1 on the surface or are induced to express it upon exposure to an agonist such as 5-OP-RU. In this regard, it is worth noting that B16F10 is one of the tumor cell lines which strongly upregulate MR1 upon 5-OP-RU exposure, thus facilitating early activation of MAIT cells. In parallel, it is of prime importance to identify MR1-depdendent T cells other than MAIT cells in mice, as these MR1-dependent T cells may play a pivotal role (s) in tumor immunity, as shown in humans (see below). Thus, interpretation of the results from MR1-knockout mice should be more cautious when taking these into account. 

Although MAIT cells express killer-cell-associated receptors, which also play a pivotal role in tumor immunity, the expression varies from one molecule to another. CD96, CD100, CD161, and CD352 (SLAMF6) are found in all MAIT cells, whilst the expression of natural cytotoxicity receptors (NCR) and receptors of the killer-cell Ig-like receptor (KIR) family is rare. Intriguingly, CD314 (NKG2D) is found in >80 % of colon MAIT cells, whereas only ~10 % of hepatic MAIT cells expressed NKG2D, suggesting that the function of MAIT cells varies from one tissue to another [40]. 

Since MAIT cells exert cytotoxic activity against bacteria-infected tumor cells in a NKG2D and CD161-independent manner, but in an MR1-dependent manner [41], it has yet to be deciphered whether MAIT cells kill intact (not bacterially infected) tumor cells; should this be the case, which and how killer-cell-associated receptors engage in cytotoxic activity should be determined.

## 8. iPSC-Derived MAIT Cells (m-reMAIT Cells) in Murine Cancer Models

**Figure 2:**
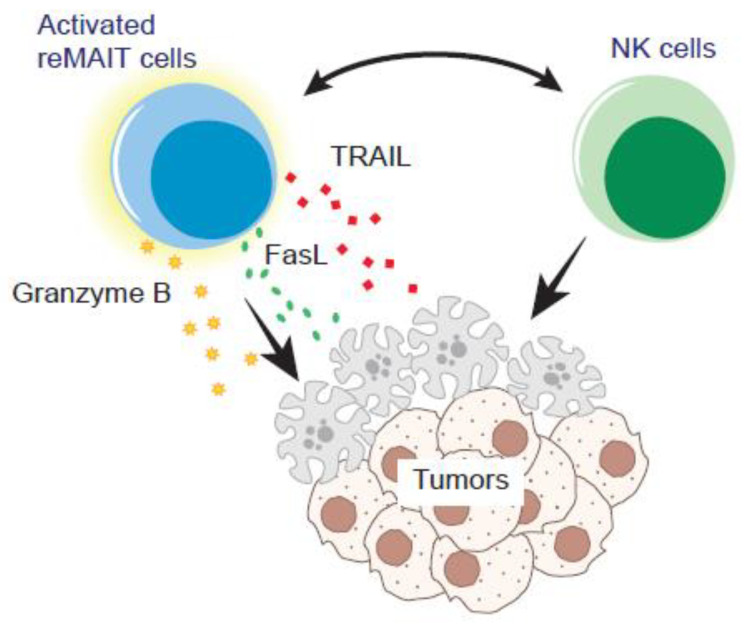
Tumor control by reMAIT cells. reMAIT cells (m-reMAIT cells and/or human iPSC-derived MAIT-like cells) could directly lyse the tumor cells and/or enhance the anti-tumor functions of NK cells. Interaction with NK cells induces *Grzmb*, *Fasl*, and *Tnfsf10*, the effector molecules pertinent to exerting the cytotoxicity in reMAIT cells in vitro. NK cells and reMAIT cells synergistically enhance tumor-killing activity in vitro and likely in vivo.

Although the above studies have implied that the role of MAIT cells is rather adjunctive in tumor immunity, our recent study has indicated that MAIT cells play a pivotal role in tumor immunity in a TCR-independent manner (Figure 2) [6]. We have shown that murine iPSC-derived MAIT cells (referred as m-reMAIT cells) exert cytotoxic activity against the NK-sensitive lymphoma cell line Yac-1 and the NK-resistant Lewis lung carcinoma (LLC) cell line in the absence of 5-OP-RU. Moreover, the combination of NK cells and m-reMAIT cells enhanced cytotoxicity against the tumor cell lines. Accordingly, adoptive transfer of m-reMAIT cells prior to tumor inoculation protected mice from metastasis formation, resulting in prolonged survival in an m-reMAIT-cell-dose dependent manner. Moreover, in vivo depletion of NK cells prior to tumor inoculation abolished the anti-tumor effects of m-reMAIT cells, further underpinning the importance of NK cells in tumor immunity. Coculture of m-reMAIT cells with NK cells resulted in enhanced transcription of *Grzmb*, *Fasl*, and *Tnfsf10* in m-reMAIT cells, suggesting that NK cells are responsible for boosting the cytotoxic activity of m-reMAIT cells. *Il17a* and *Il6*, both of which possess a dichotomic role in tumor immunity were also increased [42,43], further indicating that NK cells bolstered the anti-tumor function of m-reMAIT cells. However, *Prf-1* (transcript for perforin) was not induced in NK cells upon coculture, as seen in another report [39]. This may indicate that the increase in perforin-expressing NK cells, upon 5-OP-RU and CpG challenge, reflects indirect activation caused by an increase in MAIT cell number rather than the direct activation provoked by MAIT cells per se [39]. In this respect, it is of importance to identify the (immune) cells that mediate the interaction between MAIT cells and NK cells. 

Given that LLC does not upregulate MR1 upon 5-OP-RU challenge, the above results rather indicate the presence of MR1–TCR-independent immune pathways for MAIT cells to exert anti-tumor functions in tumor immunity. Whereas the tumor-MR1-dependent suppression of NK cell functions, such as repression of IFN-γ, is attributed to MAIT cells, MAIT-cell-dependent activation of NK cells in the presence and/or absence of 5-OP-RU has been reported [6,36,38,39]. Moreover, while a simple increase in MAIT cells appears not to be sufficient to confer anti-tumor effects [38], adoptive transfer of m-reMAIT cells is, without 5-OP-RU challenge [6]. These enigmas should be addressed in the near future. 

## 9. Therapy for Cancer with reMAIT Cells

**Figure 3:**
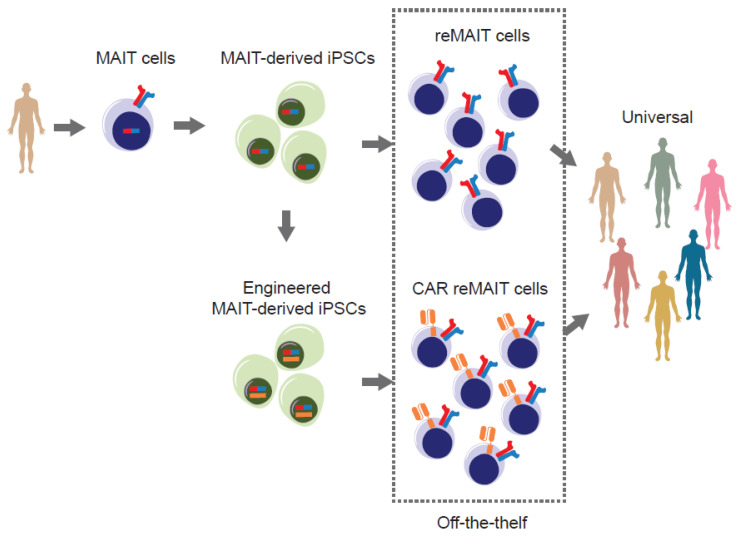
Strategy for harnessing the power of iPSC to develop off-the-shelf tumor immune therapy. MAIT cells from the healthy donor(s) are reprogrammed to iPSCs without genetic modification. MAIT-cell-derived iPSCs (MAIT-derived iPSC) are redifferentiated into MAIT-like cells (reMAIT cells) and used for tumor immune cell therapy. Alternatively, MAIT-cell-derived iPSCs are genetically engineered to express chimeric antigen receptor(s) (CAR) recognizing tumor antigen(s) and redifferentiated into MAIT-like cells expressing CAR (CAR-reMAIT cells) and used for tumor immune cell therapy. In both cases, these cells are suitable for off-the-shelf therapy because MAIT cells from different individuals show an equal reactivity to ligands and are reluctant to be rejected and/or to cause graft-versus-host disease.

As is the case for other innate-like T cells, MAIT cells have been proposed as good candidates for implementing ‘off-the-shelf’ immune therapy for cancer (Figure 3) [44]. The rationale is based on the following:

1. MAIT cells (more precisely m-reMAIT cells) exert cytotoxic activity against tumor cells without an agonist. MAIT-like cells can be differentiated from human iPSCs (referred as reMAIT cells) [45]. This ensures the preparation of a theoretically unlimited number of reMAIT cells for cell therapy.
2. MR1, the molecule responsible for restricting MAIT cell development and proliferation, is monomorphic, unlike MHC I and II, which are polymorphic in nature. Such a feature ensures that the responsiveness of TCR is equivalent among individuals, whereas that of conventional T cells varies from one person to another.
3. MAIT cells are reluctant to cause graft-versus-host disease (GvHD) and are cancer-drug resistant. These features add more value to the immune cell therapy, in that allogenic MAIT cells could be used as universal cells for adoptive transfer together with anti-cancer drugs [1,46]. Indeed, in anthracycline-treated cancer patients, MAIT cell number remains intact after drug regimen, while many, if not all, conventional T cells are eliminated. The drug-resistance of MAIT cells is conferred by the presence of a multidrug efflux pump such as CD243.
4. MAIT cells could serve as an adjuvant for bolstering anti-tumor immunity by reinforcing expansion and the effector functions of NK cells.

The above features position MAIT cells and/or reMAIT cells as one of the most promising candidates for implementing cell therapy. Although similar iPSC-based allogenic cell therapy has been proposed for iNKT cells and several clinical trials targeting iNKT cells are now underway [44], it has yet to be determined which cells are more suitable for therapeutic purposes in human. In this regard, it is worth remembering that iNKT cells are more abundant than MAIT cells in mice, but the inverse is true in humans. 

### 9.1. Future Directions Towards Designer MAIT Cells

Although MR1 constrains the development and expansion of MAIT cells harboring TRAV1-2-TRAJ33 (J12 and J20 in human), other MR1-dependent T (MR1-T) cells, which expand upon coculture with tumor cells, exist [47]. Such MR1-T cells harbor TRAV38.2/DV8-TRAJ31 and TRBV25.1-TRBJ2.3 for the α and β chains, respectively. As expected from the V–J usage, such MR1-T cells fail to recognize 5-OP-RU-loaded MR1-tetramer, however, MR1 expression on the tumor cells is required for MR1-T cells to be recognized. Importantly, transduction of MR1-T-cells-specific receptors into conventional T cell from melanoma patients redirects the T cells to recognize autologous melanoma. Given that MR1-T cells lyse the different tumor cell lines but not healthy cells, MR1-T cells could also be good candidates for immune cell therapy in cancer. It is tempting to suggest that ectopic expression of TCR for MR1-T cells in MAIT cells could confer much stronger anti-tumor activity. 

Introduction of chimeric antigen receptors to MAIT cells and/or reMAIT cells would result in another category of CAR-T cells (CAR-MAIT or CAR-reMAIT cells), as in the case for CAR-NK cells [44,48]. It is also expected that combination of MAIT cells and/or reMAIT cells expressing different CARs (recognizing different tumor antigen(s)) would exert much stronger anti-tumor functions relative to MAIT cells or reMAIT cells alone. It is worth noting that the advent of CAR-MAIT cells from peripheral blood has made it possible to open up an approach to future cell therapy. In fact, CAR-MAIT cells designed to target human EGF receptors (HER) and CD19 could kill, respectively, the breast cancer cell line MDA-231 ectopically expressing HER2, and T2 and NALM6 lymphoma cell lines expressing endogenous CD19 [49]. Remarkably, the cytotoxic activity of CAR-MAIT cells was equivalent to that of CAR-CD8 T cells and produced less IFN-γ and GM-CSF than CAR-CD8 T cells. Given that GM-CSF inhibition mitigates the cytokine release syndrome and neuroinflammation inherent to CAR-T therapy and enhances CAR-T function [50], it is tempting to expect that CAR-reMAIT cells would exert a similar activity. Thus far, CAR-reMAIT cells hold a promising future for the implementation of allogenic cell therapy and a foundation for realizing off-the-shelf medication.

However, since the tumor microenvironment is often hostile for immune cells exerting anti-tumor functions, one must consider how to overcome the immune-suppressive conditions in tumors before developing the above therapies. The first criterion is to determine whether the functions of MAIT cells in situ (within the tumor) are perturbed or not. This can be assessed by analyzing cytokine-production ability and/or cytotoxic activity against tumor cells. Phenotypical analysis by flow cytometry would reveal whether such MAIT cells exhibit an exhausted phenotype harboring enhanced expression of PD-1 and/or CTLA-4, the immune checkpoint molecules, as is the case for CD8 T cells; should this be the case, the use of ICHs would be beneficial. It should be emphasized that MAIT cells are ubiquitously found in TILs as a major component of Tc17 cells across different tumors [2]. Intriguingly, the frequency of Tc17 cells and/or MAIT cells positively correlates with responsiveness to anti-PD-1 therapy in melanoma patients, further supporting the possibility that MAIT cells are anti-tumor in nature and that ICHs could reinvigorate the compromised functions of MAIT cells in the tumor microenvironment [51,52]. Regardless of such utility in solid tumors, other obstacles inherent of the tumor microenvironment, such as cancer-associated fibroblasts (CAF), must be conquered in the fight against tumors. Although intratumor injection of CAR-MAIT cells and/or CAR-reMAIT cells is envisioned in combination with ICHs, it is currently unknown whether such a combination would be sufficient to suppress tumor expansion and metastasis. 

### 9.2. Issues to Be Addressed in the near Future

The following issues should be addressed and clarified to ensure that MAIT cell use is reasonable way to exploit future cell therapy for tumors.

1. While m-reMAIT cells kill Yac-1 and LLC, whether reMAIT cells (human iPSCs-derived MAIT-like cells) exert cytotoxic activity against an array of human tumor cells, as MR1-T cells do, should be determined. In this case, it is also important to determine whether the cytotoxic activity is dependent on 5-OP-RU or not, in other words, TCR-dependent or independent.
2. Whether m-reMAIT cells and reMAIT cells have an anti-tumor effect in therapeutic models should be addressed. Present data only demonstrate that adoptive transfer of m-reMAIT cells prior to tumor inoculation inhibits tumor growth and prolongs mouse survival. It is thus imperative to examine whether adoptive transfer of m-reMAIT cells and/or reMAIT cells after tumor inoculation also engenders inhibition of tumor expansion (therapeutic model).
3. Whether CAR-MAIT cells and/or CAR-reMAIT cells exert much stronger anti-tumor effects, including cytotoxic activity, than MAIT cells and/or reMAIT cells in both prophylaxis and therapeutic models should also be interrogated.
4. Since MAIT cells are thought not to induce GvHD, it is necessary to examine whether this is also applicable to CAR-MAIT cells and/or CAR-reMAIT cells.
5. As CAR-T cells could last for more than a decade after transfer into humans [53], it is also compulsory to examine how long CAR-MAIT cells and/or CAR-reMAIT cells survive in highly immunocompromised mice, such as NOG or NSG mice.

The above knowledge would pave the way for implementing tumor cell therapy with reMAIT cells and/or CAR-reMAIT cells.

## Data Availability

Not applicable.
